# Switching to brolucizumab: injection intervals and visual, anatomical and safety outcomes at 12 and 18 months in real-world eyes with neovascular age-related macular degeneration

**DOI:** 10.1186/s40942-023-00445-0

**Published:** 2023-02-01

**Authors:** Joseph M. Coney, Ryan Zubricky, Samriddhi Buxy Sinha, Nina Sonbolian, Lujia Zhou, Thomas P. Hull, Shawn A. Lewis, David G. Miller, Michael A. Novak, Scott D. Pendergast, Hang Pham, Sean M. Platt, Llewelyn J. Rao, Jerome P. Schartman, Lawrence J. Singerman, Richard Donkor, Margaret Fink, Jasmyne McCoy, Helene Karcher

**Affiliations:** 1 Retina Associates of Cleveland Inc, 24075 Commerce Park, Beachwood, OH 44122 USA; 2 Geisinger Eye Institute, Danville, PA USA; 3 Novartis AG, Basel, Switzerland; 4 KMK Consulting, Morristown, NJ USA

**Keywords:** Anti-VEGF, Brolucizumab, Beovu, Injection intervals, nAMD, Neovascular age-related macular degeneration, Switch patients, Treatment burden

## Abstract

**Background:**

The anti-vascular endothelial growth factor (anti-VEGF) injection interval influences treatment burden and compliance in neovascular age-related macular degeneration (nAMD). This real-world study investigates visual acuity (VA), injection-interval extension, central macular thickness (CMT) and safety in nAMD eyes switched to the anti-VEGF agent brolucizumab and followed for up to 18 months.

**Methods:**

This retrospective study included patients with nAMD who were switched from other anti-VEGF agents to brolucizumab only. Patient eyes were grouped into three nested cohorts with the overall cohort receiving ≥ 1 brolucizumab injection, the second receiving ≥ 3 brolucizumab injections with a follow-up period of ≥ 12 months and the third cohort receiving ≥ 3 brolucizumab injections with a follow-up period of ≥ 18 months. Study endpoints included changes from baseline at 12 or 18 months in VA, injection intervals, and CMT. Sub-group analyses were conducted using baseline injection interval length or baseline VA as qualifiers.

**Results:**

Overall, 482 eyes received ≥ 1 brolucizumab injection; 174 eyes received ≥ 3 brolucizumab injections with ≥ 12 months of follow-up, and 95 eyes received ≥ 3 brolucizumab injections with ≥ 18 months of follow-up. VA (mean [95% confidence intervals]) remained stable relative to baseline after 12 months (− 1.1 [− 3.7, 1.6] letters; p = 0.42) and 18 months (0.0 [− 3.1, 3.1] letters; p = 0.98) of brolucizumab treatment, respectively, and pre-switch injection intervals or baseline VA had no notable effect. Following the switch to brolucizumab, injection intervals were extended from baseline to month 12 by 26.9 (19.7, 34.0) days (p < 0.0001), and eyes with pre-switch injection intervals < 8 weeks were able to have their injection intervals extended by 23.6 days longer than eyes with pre-switch injection intervals ≥ 8 weeks. At 18 months, injection intervals were extended by 36.3 (25.6, 46.9) days (p < 0.0001) compared to baseline. Following switch to brolucizumab, CMT was reduced at both 12 and 18 months (12 months: − 35.2 (− 51.7, − 18.8) µm, p < 0.0001; 18 months: − 38.9 (− 54.3, − 22.0) µm, p < 0.0001). Intraocular inflammation-related adverse events were reported in 4.6% of brolucizumab-treated eyes.

**Conclusions:**

This real-world study demonstrates that injection intervals may be significantly extended with maintained vision and reduced CMT in nAMD eyes switching to brolucizumab therapy from other anti-VEGFs.

**Supplementary Information:**

The online version contains supplementary material available at 10.1186/s40942-023-00445-0.

## Background

Age-related macular degeneration (AMD) is a degenerative eye disease and a leading cause of permanent visual impairment in older adults [1]. Neovascular exudative AMD (nAMD) is an advanced form of AMD that is characterized by macular neovascularization and the build-up of subretinal and intraretinal fluid, which can be quantified by optical coherence tomography (OCT) imaging of the central macular thickness (CMT) [2, 3].

Anti-vascular endothelial growth factor (anti-VEGF) agents have become standard of care for the treatment of nAMD [4]. Brolucizumab is a single-chain antibody fragment that rapidly penetrates the retina and inhibits all isoforms of VEGF-A [5]. In current clinical practice, brolucizumab (6 mg) is indicated for the treatment of nAMD with post-loading phase ocular injection intervals of 8–12 weeks [6, 7]. In the pivotal HAWK and HARRIER studies, which evaluated functional and anatomical outcomes in treatment-naive nAMD patients following anti-VEGF injection every 8 or 12 weeks, brolucizumab provided similar vision gains and superior reduction in CMT compared with aflibercept, with most brolucizumab-treated patients remaining on a 12-week dosing interval at week 48 [8]. The visual acuity (VA) gains and anatomical improvements achieved with brolucizumab in HAWK and HARRIER at week 48 were sustained at week 96 [9].

In nAMD, the anti-VEGF injection interval is an important factor in treatment compliance and treatment burden. Frequent intravitreal injections with anti-VEGF agents is often burdensome for patients and physicians, and under-treatment, due to non-adherence to a particular treatment regimen, puts the patient at risk of avoidable vision loss [10]. Variations in dosing regimens such as treat-and-extend (T&E) have therefore been implemented to balance adequate disease control with fewer anti-VEGF injections over time by extending the injection interval lengths, which reduces the overall injection burden [11, 12]. Recent small-scale real-world studies in Japan have reported improved anatomical outcomes, injection intervals of up to 14 weeks, and either maintained or improved VA after switching from other anti-VEGFs to brolucizumab [13, 14]. The aim of this real-world study was to investigate VA, injection interval extensions, CMT and safety in nAMD patients who were switched from another anti-VEGF to treatment with brolucizumab only for up to 18 months.

## Methods

### Study cohorts

**Fig. 1:**
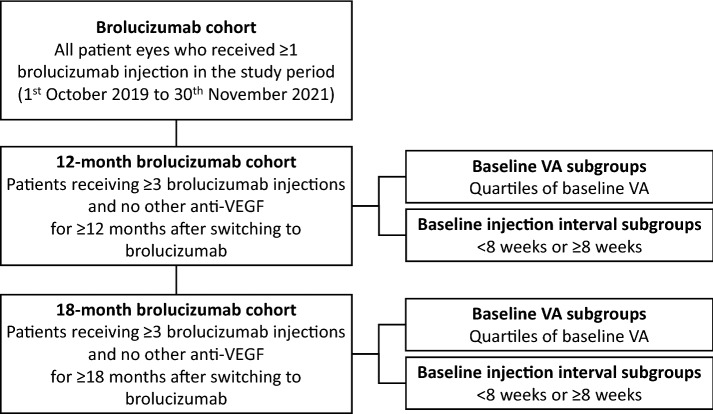
Patient selection and definition of study cohorts and sub-groups

This was a retrospective real-world study of nAMD patients treated with brolucizumab 6 mg as per label [6, 7] between 1st October 2019 and 30th November 2021 (i.e., the study period) at a large retinal practice located in Cleveland, Ohio, USA. Anonymized patient data was extracted on 1st Dec 2021 that included all patient eyes who received 1 or more brolucizumab injections during the study period (the brolucizumab cohort). A 12-month brolucizumab cohort (derived from the brolucizumab cohort) included all nAMD patient eyes who were switched to brolucizumab from other anti-VEGFs, had at least 12 months of follow-up after the first brolucizumab injection, and had received ≥ 3 brolucizumab injections and no other anti-VEGF agent within the first 12 months after switching to brolucizumab (Fig. 1). These criteria were set to ensure that this study provides an analysis of those patients who have remained on brolucizumab treatment for a notable period of time (12 months). An 18-month brolucizumab sub-cohort was derived from the 12-month brolucizumab cohort and consisted of patient eyes with at least 18 months of follow-up after the first brolucizumab injection and that were treated with no other anti-VEGF during these 18 months. Sub-group analyses were carried out in the 12-month brolucizumab cohort and in the 18-month brolucizumab cohort using pre-switch injection interval length and pre-switch VA as qualifiers.

### Clinical assessments and treatment decisions

Patients were assessed and treated at one of the Retina Associates of Cleveland, Inc. (RACI) clinic sites. At each visit, patients’ VA was measured with a Snellen chart (converted to Early Treatment Diabetic Retinopathy Study [ETDRS] letters for analysis) and intraocular pressure was checked along with a complete ophthalmic examination using dilated slit lamp biomicroscopy. CMT was measured using a Heidelberg OCT Spectralis (model no. 5244; software version: Heidelberg eye-explorers V1.10.4.0), a Zeiss FP-7 M (model no. FF4; software version: New vision fundus V3.2) or a Zeiss OCT Angioplex (model no. 5000; software version: V11.5.2.54532). Treatment interval extensions were based on the extent of drying of retinal fluid. The shortest interval between brolucizumab injections in the study cohort was 20 days.

### Study endpoints

Baseline VA, injection intervals, and CMT were collected and analyzed at the time of the first brolucizumab injection, and after 12 and 18 months of follow-up, and reported as changes relative to baseline measurements. VA and CMT at Month 12 and at Month 18 were defined as the measurements collected closest (± 90 days) to the injections at the corresponding months. Missing values for CMT were defined as measurements not collected at baseline or measurements collected outside of the ± 90-day window at Month 12 and Month 18, respectively. Eyes with ‘light perception’ vision were excluded from the VA analysis, as it was not possible to convert ‘light perception’ into ETDRS letters [15]. The baseline injection interval was defined as the time between the last pre-switch anti-VEGF injection and the first brolucizumab injection. The injection intervals at Month 12 and at Month 18 were defined as the time between the injection closest to the respective month and the preceding injection. Safety data collected included all intraocular inflammation (IOI) events, retinal vasculitis (RV), retinal vascular occlusion (RO) in the presence or absence of IOI and secondary to brolucizumab use.

### Sub-group analyses

Baseline injection interval sub-group analyses were conducted to investigate the effect of baseline injection interval length (i.e., < 8 weeks or ≥ 8 weeks) on VA, injection intervals, and CMT at Months 12 and 18. The injection interval cut-off of 8 weeks was chosen as it provided reasonably well-balanced sub-groups.

Baseline VA sub-group analyses, where patient eyes were grouped into four quartiles based on baseline VA, were conducted to evaluate the effect of baseline VA on VA, injection intervals, and CMT at Months 12 and 18.

### Statistical methods

Outcome measures were calculated by subtracting the baseline value from the values at Month 12 or Month 18, respectively, and changes in VA, injection intervals, and CMT are reported as mean ± standard deviation (SD) or mean (95% confidence intervals [CI]), with the patient eye as the unit of analysis. Outcomes were analyzed using a mixed model to account for any inter-eye correlation. Statistical significance was determined by t-test analyses using R software, and P values < 0.05 were considered statistically significant. A missing value analysis was performed by calculating the percentage of missing values at baseline, Month 12, and Month 18. VA was converted from Snellen numerators, denominators and logMAR values to ETDRS letters as described by Gregori et al.[16]

## Results

### Baseline descriptive analysis

A total of 482 eyes from 414 patients received ≥ 1 brolucizumab injection in the study period and included the 174 eyes from 154 patients receiving ≥ 3 brolucizumab injections who made up the 12-month brolucizumab cohort (Table 1). A further 6 eyes received only 1 or 2 brolucizumab injections in the study period and hence were excluded from the analysis despite having the full 12 months of follow up. The reasons for not continuing on brolucizumab included lost to follow-up, decision to switch back to previous anti-VEGF after benefit/risk discussion, AE or complication reported with brolucizumab or the patient was a poor responder (i.e. unable to extend longer than previous treatment interval). The mean (SD) patient age in the 12-month brolucizumab cohort was 80.5 (7.7) years and 55.8% of patients were females. Eyes in the 12-month brolucizumab cohort had mean pre-switch VA of 60.8 (17.1) ETDRS letters and mean pre-switch CMT of 292.2 (113.3) μm. A total of 120/174 (69.0%) eyes had pre-switch anti-VEGF injection intervals < 8 weeks and 54/174 (31.0%) eyes had pre-switch injection intervals ≥ 8 weeks.

**Table 1:** Clinical and baseline characteristics of the study 12-month brolucizumab cohort

	12-month brolucizumab cohort		Baseline injection interval sub-groups		Baseline VA sub-groups (ETDRS letter range)			
	Patients N = 154	Eyes N = 174	Injection intervals < 8 weeks n = 120 eyes	Injection intervals ≥ 8 weeks n = 54 eyes	Quartile 1 (74.2, 85.0) n = 43 eyes	Quartile 2 (65.1, 74.2) n = 40 eyes	Quartile 3 (53.9, 65.1) n = 48 eyes	Quartile 4 (19.9, 53.9) n = 43 eyes
Age, years (Mean, [SD])	80.5 (7.7)	–	80.8 (7.6)	80.7 (8.6)	78.3 (9.0)	80.4 (7.0)	81.6 (7.5)	82.5 (7.5)
Gender								
Female: N, %	86 (55.8)	–	73 (60.8)	25 (46.3)	27 (62.8)	23 (57.5)	26 (54.2)	22 (51.2)
Male: N, %	67 (43.5)	–	46 (38.3)	29 (53.7)	15 (34.9)	17 (42.5)	22 (45.8)	21 (48.8)
Time from nAMD diagnosis to first brolucizumab (mean months [SD])	–	47.7 (35.5)	–	–	–	–	–	–
Time from first anti-VEGF to first brolucizumab (mean months [SD])	–	39.0 (33.3)	–	–	–	–	–	–
VA (ETDRS letters; mean [SD])	–	60.8 (17.1)	61.9 (16.3)	58.5 (18.7)	78.9 (3.3)	69.4 (2.5)	60.3 (3.6)	35.4 (10.2)
Injection interval (mean days [SD])	–	47.8 (25.1)	36.6 (7.7)	72.8 (31.6)	43.9 (14.2)	50.8 (34.1)	48.9 (28.5)	47.7 (19.5)
Follow up period from first brolucizumab injection (mean days [SD])	–	562.8 (104.8)	571.9 (107.6)	542.7 (96.3)	612.1 (86.3)	569.1 (113.3)	544.6 (96.7)	528.1 (106.1)
CMT (N [%])	–	162 (93.1)	115 (95.8)	47 (87.0)	42 (97.7)	35 (87.5)	45 (93.8)	40 (93.0)
CMT, µm (Mean [SD])	–	292.2 (113.3)	294.8 (111.6)	285.8 (118.4)	265.8 (74.6)	287.3 (66.9)	280.0 (70.1)	338.0 (186.0)
IRF (n [%])	–	40 (23.0)	23 (19.2)	17 (31.5)	5 (11.6)	6 (15.0)	12 (25.0)	17 (39.5)
SRF (n [%])	–	102 (58.6)	68 (56.7)	34 (63.0)	23 (53.5)	23 (57.5)	31 (64.6)	25 (58.1)
PED (n [%])	–	109 (62.6)	75 (62.5)	34 (63.0)	18 (41.9)	25 (62.5)	37 (77.1)	29 (67.4)

*CMT* central macular thickness, *ETDRS* Early Treatment Diabetic Retinopathy Study, *IRF* intraretinal fluid, *nAMD* neovascular age-related macular degeneration, *PED* pigment epithelial detachment, *SD* standard deviation, *SRF* subretinal fluid, *VA* visual acuity, *VEGF* vascular endothelial growth factor

The 18-month brolucizumab cohort (Additional file 1: Table S1) included 95 eyes from 85 patients. The mean patient age in this sub-cohort was 80.0 (7.6) years and 57.7% were female. Eyes had mean pre-switch VA of 64.7 (15.9) ETDRS letters and mean CMT of 283.7 (76.2) μm. A total of 69/95 (72.6%) eyes in this sub-cohort had pre-switch injection intervals < 8 weeks, and 26/95 (27.4%) eyes had pre-switch injection intervals ≥ 8 weeks. Baseline demographics and clinical characteristics of the brolucizumab cohort were similar to those of the 12-month brolucizumab cohort.

CMT data were missing for 12/174 (6.9%) eyes in the 12-month brolucizumab cohort and from 6/95 (6.3%) eyes in the 18-month brolucizumab cohort. Two patients were excluded from the 12-month VA analysis, as they only had light perception VA at Month 12.

### Effects of switching to brolucizumab on vision

Average vision across the cohort remained stable at 12 months of brolucizumab treatment after switching, with the cohort experiencing a mean change from baseline of − 1.1 (− 3.7, 1.6) ETDRS letters (p = 0.42), without any notable effect of the pre-switch injection interval (eyes with pre-switch injection interval < 8 weeks: − 1.7 [− 4.9, 1.5] ETDRS letters [p = 0.30]; eyes with pre-switch injection interval ≥ 8 weeks: 0.3 [− 4.4, 4.9] ETDRS letters [p = 0.90]. VA change at Month 12 in the pre-switch VA quartiles was − 3.7 (− 7.3, − 0.1) ETDRS letters (p = 0.04) in quartile (Q) 1 (the quartile with the best VA), −.0 (−.7, 2.7) ETDRS letters (p = 0.39) in Q2, − 1.6 (− 4.9, 1.8) ETDRS letters (p = 0.37) in Q3, and + 3.0 (− 3.3, 9.2) ETDRS letters (p = 0.35) in Q4 (the quartile with the worst baseline VA). Similar results for the change in VA were observed in the 18-month brolucizumab cohort (Table 2).

**Table 2:** VA change from baseline at 12 and 18 months after switching to brolucizumab

Cohort	VA change from baseline mean (95% CI) ETDRS letters	*P*-value
12-month brolucizumab cohort (N = 174 eyes)*	− 1.1 (− 3.7, 1.6)	0.42
Baseline injection interval sub-groups		
< 8 weeks ( n = 120 eyes)	− 1.7 (− 4.9, 1.5)	0.30
≥ 8 weeks (n = 54 eyes)	0.3 (− 4.4, 4.9)	0.90
Baseline VA sub-groups (ETDRS letter range)		
Eyes with the best baseline VA (74.1–85.0 letters; n = 43 eyes)	− 3.7 (− 7.3, − 0.1)	0.04
Eyes with better baseline VA (65.1–74.2 letters; n = 40 eyes)	− 2.0 (− 6.7, 2.7)	0.39
Eyes with worse baseline VA (53.9–65.1 letters; n = 48 eyes)	− 1.6 (− 4.9, 1.8)	0.37
Eyes with the worst baseline VA (19.9–53.9 letters; n = 43 eyes)	+ 3.0 (− 3.3, 9.2)	0.35
ETDRS letter vision change from baseline at Month 12	n (%)	
≥ 30 letters gained	3 (1.7)	–
≥ 15 to < 30 letters gained	13 (7.5)	–
≥ 5 to < 15 letters gained	26 (14.9)	–
< 5 letters lost to < 5 letters gained	94 (54.0)	–
≥ 5 to < 15 letters lost	19 (10.9)	–
≥ 15 to < 30 letters lost	7 (4.0)	–
≥ 30 letters lost	10 (5.7)	–

	VA change from baseline mean (95% CI) ETDRS letters	*P*-value
18-month brolucizumab cohort (N = 95 eyes)**	0.0 (− 3.1, 3.1)	0.98
Baseline injection interval sub-groups		
< 8 weeks (n = 69 eyes)	− 0.1 (− 3.3, 3.0)	0.94
≥ 8 weeks (n = 26 eyes)	0.2 (− 8.0, 8.3)	0.97
Baseline VA sub-groups (ETDRS letter range)		
Eyes with the best baseline VA (76.2–85.0 letters; n = 21 eyes)	− 5.5 (− 10.0, -0.9)	0.02
Eyes with better baseline VA (67.1–76.2 letters; n = 27 eyes)	− 2.6 (− 5.6, 0.4)	0.08
Eyes with worse baseline VA (58.8–67.1 letters; n = 23 eyes)	1.8 (− 1.6, 5.1)	0.29
Eyes with the worst baseline VA (19.9–58.8 letters; n = 24 eyes)	5.9 (− 3.0, 14.8)	0.18
ETDRS letter vision change from baseline at Month 18	n (%)	
≥ 30 letters gained	2 (2.1)	–
≥ 15 to < 30 letters gained	8 (8.4)	–
≥ 5 to < 15 letters gained	12 (12.6)	–
< 5 letters lost to < 5 letters gained	47 (49.5)	–
≥ 5 to < 15 letters lost	20 (21.1)	–
≥ 15 to < 30 letters lost	3 (3.2)	–
≥ 30 letters lost	3 (3.2)	–

*CI* confidence interval, *ETDRS* Early Treatment Diabetic Retinopathy Study, *VA* visual acuity

^*^all nAMD patients who switched to brolucizumab from other anti-VEGFs, had at least 12 months of follow-up after the first brolucizumab injection and received ≥ 3 brolucizumab injections and no other anti-VEGF agent within the first 12 months of therapy

^**^all nAMD patients who switched to brolucizumab from other anti-VEGFs, had at least 18 months of follow-up after the first brolucizumab injection and received ≥ 3 brolucizumab injections and no other anti-VEGF agent within the first 18 months of therapy

### Effects of switching to brolucizumab on injection interval lengths

**Fig. 2:**
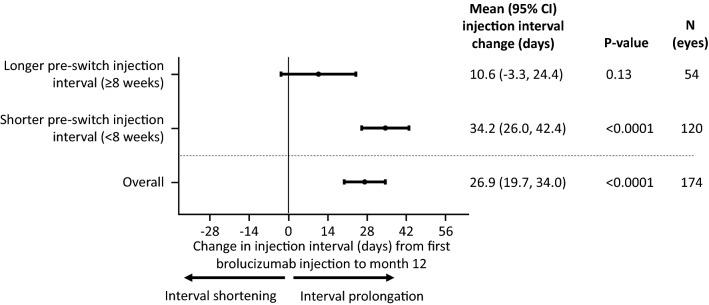
Effect of baseline injection interval length on injection interval length at Month 12. CI, confidence interval

Twelve months after switching to brolucizumab, a mean (95% CI) injection interval extension relative to baseline of + 26.9 (19.7, 34.0) days (i.e., + 3.8 weeks; p < 0.0001) was observed. Eyes with pre-switch injection intervals < 8 weeks experienced a greater (but not statistically significant) mean post-switch injection interval extension from baseline at Month 12 (+ 34.2 [26.0, 42.4] days [i.e., + 4.9 weeks]; p < 0.0001) compared to eyes with pre-switch injection intervals of ≥ 8 weeks (+ 10.6 [− 3.3, 24.4] days [i.e., + 1.5 weeks]; p = 0.13; Fig. 2). Similar results were found in the 18-month brolucizumab cohort (Additional file 2: Figure S1).

**Fig. 3:**
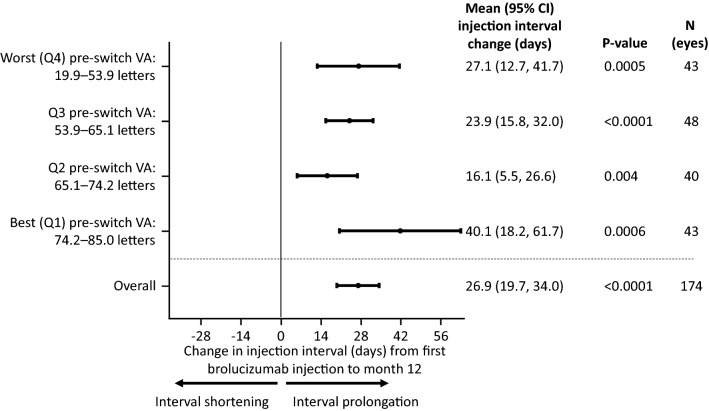
Effect of baseline VA on injection interval length at Month 12. CI, confidence interval; Q, quartile; VA, visual acuity

The pre-switch VA sub-group analysis revealed that the injection interval extension at Month 12 was independent of pre-switch VA (Fig. 3). The mean interval extensions were 40.1 (18.2, 61.7) days (i.e., 5.7 weeks) in Q1 (the quartile with the best pre-switch VA), 16.1 (5.5, 26.6) days (i.e., 2.3 weeks) in Q2, 23.9 (15.8, 32.0) days (i.e., 2.2 weeks) in Q3 and 27.1 (12.7, 41.7) days (i.e., 1.8 weeks) in Q4 (the quartile with the worst pre-switch VA). Similar patterns were observed in the 18-month brolucizumab cohort (Additional file 3: Figure S2).

### Effects of switching to brolucizumab on CMT

**Fig. 4:**
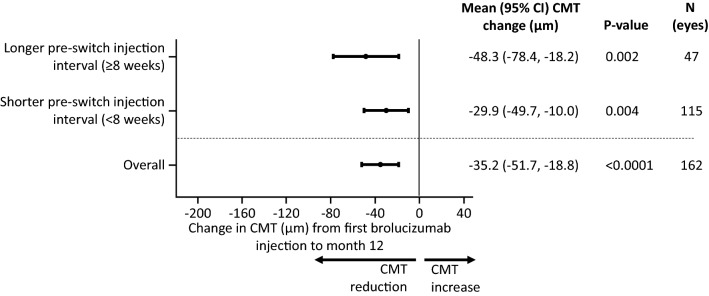
Effect of baseline injection interval length on CMT at Month 12. CI, confidence interval; CMT, central macular thickness; VA, visual acuity

**Fig. 5:**
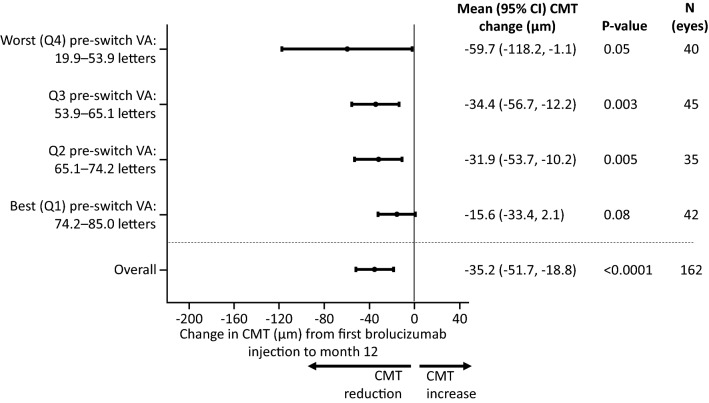
Effect of baseline VA on CMT at Month 12. CI, confidence interval; CMT, central macular thickness; Q, quarter; VA, visual acuity

Mean CMT was reduced by − 35.2 [− 51.7, − 18.8] µm compared to the pre-switch CMT (p < 0.0001) 12 months after switching to brolucizumab. In patients with pre-switch injection intervals of < 8 weeks, mean CMT reduced by − 29.9 [− 49.7, − 10.0] µm [p = 0.004] relative to baseline compared with − 48.3 [− 78.4, − 18.2] µm [p = 0.002] in those with injection intervals ≥ 8 weeks (Fig. 4). Greater mean reductions in CMT were observed in those with lower pre-switch VA (Q1 [the quartile with the best pre-switch VA]: − 15.6 [− 33.4, 2.1] µm [p = 0.08]; Q2: -31.9 [-53.7, -10.2] µm [p = 0.005]; Q3: − 34.4 [− 56.7, − 12.2] µm [p = 0.003]; Q4 [the quartile with the worst baseline VA]: − 59.7 [− 118.2, − 1.1] µm [p = 0.05]; Fig. 5). Comparable reductions in CMT (− 38.9 [− 54.3, − 22.0] µm compared to the pre-switch CMT [p < 0.0001]) were observed in the 18-month brolucizumab cohort (Additional file 4: Figure S3; Additional file 5: Figure S4).

### Safety

In the brolucizumab cohort (i.e., all 482 patient eyes receiving ≥ 1 brolucizumab injection during the study period) a total of 22/482 (4.6%) eyes in 21 patients experienced IOI-related AEs, including 4/482 (0.8%) eyes with concomitant RV, of which 2 eyes (2/482; 0.4%) had IOI, RV and RO. Two eyes (2/482 eyes; 0.4%) with IOI with RV experienced ≥ 15 letters vision loss (i.e., a 0.4% [2/482] overall risk of developing IOI with RV and experiencing at least moderate vision loss). One of the 2 eyes with IOI, RV and vision loss of ≥ 15 letters had concomitant RO, (i.e., a 0.2% [1/482] overall risk of developing IOI with RV and RO and experiencing at least moderate vision loss). Neither of the 2 patient eyes with IOI, RV and RO experienced vision loss of ≥ 30 letters. Most (82%; 18/22) of the inflammation events presented within the first 6 months of treatment initiation, 14% (3/22) presented between 6–12 months, and 5% (1/22) between 12–18 months.

## Discussion

In this study, mean VA was maintained 12 months after switching to brolucizumab from other anti-VEGFs, CMT was reduced by a mean of 35 µm and the injection interval was extended, particularly in eyes with high pre-switch injection burden (i.e., pre-switch injection intervals < 8 weeks). The pre-switch VA does not seem to influence the ability to extend injection intervals at Month 12 and Month 18. It is notable that these results were obtained in real-world eyes with a particularly high burden of disease. In general, these patients were switched to brolucizumab because of an incomplete response to prior anti-VEGF agents and/or their treatment interval could not be extended without increasing nAMD disease activity.

Improved anatomical outcomes without improvement in VA has also been reported in real-world switch studies involving other anti-VEGF agents, and it has been speculated that the lack of translation of anatomical gains into visual gains may result from permanent structural damage to photoreceptors caused by long-term nAMD disease duration [17].

Although the real-world patient population included in this study had a baseline VA similar to the baseline VA in the randomized, controlled HAWK and HARRIER trials, the real-world study population in our study differed from the HAWK and HARRIER nAMD patient populations in that all eyes had previously been treated with an anti-VEGF and that a T&E injection regimen was used, whereas HAWK and HARRIER included only treatment-naive eyes and used fixed dosing regimens [8, 9].

The T&E regimen facilitates the adaptation of anti-VEGF injection intervals according to nAMD disease activity, with short injection intervals being frequently used where high disease activity is evident and extended injection intervals being preferred for patients with low disease activity [10–12]. A recent US-wide study showed that over half of nAMD patients are still receiving anti-VEGFs with injection intervals ≤ 8 weeks after 2 years of treatment, which is a substantial burden for nAMD patients [18]. Brolucizumab is indicated for post-loading dose injection intervals of at least 8 weeks [6, 7]. However, this real-world data shows that it is possible for patients on pre-brolucizumab injection intervals shorter than 8 weeks to successfully switch to brolucizumab, because switched patients are likely to extend their injection intervals to ≥ 8 weeks after switching to brolucizumab.

Any treatment decision to switch a patient with nAMD to brolucizumab needs to be based on an individual benefit-risk assessment due to the associated risk of IOI-related AEs. In this study, adverse events were evaluated in all eyes that were treated with at least one brolucizumab injection during the study period (482 eyes) to avoid bias in favor of eyes that tolerate ≥ 3 brolucizumab injections over the 12-month follow-up. A total of 4.6% of brolucizumab-treated eyes experienced IOI-related AEs, and 82% of these events took place within 6 months after initiating treatment with brolucizumab. These rates are similar to those observed in a post hoc analysis of the Phase 3 HAWK and HARRIER studies of anti-VEGF-treatment-naive eyes with nAMD, where 50/1088 brolucizumab-treated eyes (4.6%) developed IOI, most frequently (74%) within 6 months after the first administration of brolucizumab [19]. Further details on the IOI-related AEs will be published in a separate manuscript. Regarding the underlying mechanism, the BASIC49 study identified an immune-mediated reaction in response to brolucizumab treatment that can cause RV and/or RO, typically in the presence of IOI. [20] However, the pathway and conditions that trigger these inflammatory events remain unclear.

A key strength of this study is that it is, to the best of our knowledge, the largest real-world study of eyes on brolucizumab for at least 12 months of follow up, which report data on the three key effectiveness outcomes of VA, interval extension, and CMT.

Other brolucizumab real-world studies include the REBA study of 105 nAMD eyes with a mean follow-up of 10.4 months [21], the SHIFT study of 63 eyes that were followed up for four weeks [22], and two Japanese studies of 68 and 45 eyes, respectively, that were followed up for 1 year [13, 14]. Additionally, although the large REALIZE study included 3501 patients that were followed for at least 1 year, only reported on injection interval outcomes and not VA nor CMT [23].

In our study, the eyes also have differing lengths of treatment history prior to starting brolucizumab, which provides a very broad, heterogeneous study population that reflects real-world treatment conditions. Additionally, the extent of missing data was low in this study for all variables, including CMT, and complete Electronic Health Records data for each eye, including which eye was treated and clinical outcomes, allowed for in-depth analyses.

Confounding factors include that the data was collected during the 2020 COVID-19 pandemic, meaning that timely patient follow-up might have been at least partially impacted by social distancing and other infection control measures implemented to reduce the risk of COVID-19 transmission. A recent study has however shown that at the practice we report data from, this effect was limited to a mean 5% reduction in clinic visits over a 10-week period during the COVID-19 pandemic (16th March to 31st May 2020) compared to a corresponding pre-pandemic 10-week period (1st January to 15th March 2020), and that the negative impact of COVID-19 measures on clinic visits disappeared by the end of the 10-week study period [24]. Based on these results, it is therefore unlikely that the COVID-19 pandemic had any meaningful impact on the overall results of our 26-month study. Other limitations include the small number of patients (< 20) for the 18-month brolucizumab cohort subgroup analyses, the limited geographical reach of this study, and the lack of a randomized control cohort.

## Conclusions

This real-world study demonstrates that nAMD eyes on anti-VEGF therapy can significantly prolong injection intervals by switching to brolucizumab therapy from other anti-VEGFs, while maintaining vision and reducing CMT. Importantly, the data show that it is possible to reduce the anti-VEGF injection burden in nAMD eyes with pre-switch injection intervals < 8 weeks, and a smaller sample with 18-month follow-up data suggests that this result observed at Month 12 is likely maintained at Month 18.

## Supplementary Information

**Additional file 1: Table S1.** Baseline characteristics for the 18-month brolucizumab cohort.

**Additional file 2: Figure S1.** Effect of baseline injection interval length on injection interval length at Month 18.

**Additional file 3: Figure S2.** Effect of baseline VA on injection interval length at Month 18.

**Additional file 4: Figure S3.** Effect of baseline injection interval length on CMT at Month 18.

**Additional file 5: Figure S4.** Effect of baseline VA on CMT at Month 18.

## Data Availability

The data that support the findings of this study are available from Retina Associates of Cleveland Inc, but restrictions apply to the availability of these data, which were used under license for the current study, and so are not publicly available. Data are however available from the authors upon reasonable request and with permission of Retina Associates of Cleveland Inc.

## Abbreviations

AMD: Age-related macular degeneration
CI: Confidence interval
CMT: Central macular thickness
COVID-19: Coronavirus disease 2019
ETDRS: Early Treatment Diabetic Retinopathy Study
IOI: Intraocular inflammation
IRF: Intraretinal fluid
logMAR: Logarithm of the Minimum Angle of Resolution
nAMD: Neovascular age-related macular degeneration
OCT: Optical coherence tomography
PED: Pigment epithelial detachment
Q: Quartile
RO: Retinal vascular occlusion
RV: Retinal vasculitis
SD: Standard deviation
SRF: Subretinal fluid
T&E: Treat-and-extend
VA: Visual acuity
VEGF: Vascular endothelial growth factor
