# Supplementary material for: Switching to brolucizumab: injection intervals and visual, anatomical and safety outcomes at 12 and 18 months in real-world eyes with neovascular age-related macular degeneration
Source: Int J Retina Vitreous. 2023 Feb 1;9:8. doi: 10.1186/s40942-023-00445-0 (PMC9891747; doi:10.1186/s40942-023-00445-0)
Supplement: Supplementary file 1 — Additional file 1: Table S1. Baseline characteristics for the 18-month brolucizumab cohort. [file 40942_2023_445_MOESM1_ESM.pdf]

Supplementary Table 1. Baseline characteristics for the 18-month brolucizumab cohort

	18-month brolucizumab cohort		Baseline injection interval sub-groups		Baseline VA sub-groups (ETDRS letter range)			
	Patients N=85	Eyes N=95	<8 weeks n=69 eyes	≥8 weeks n=26 eyes	Quartile 1 (76.2, 85.0) n=21 eyes	Quartile 2 (67.1, 76.2) n=27 eyes	Quartile 3 (58.8, 67.1) n=23 eyes	Quartile 4 (19.9, 58.8) n=24 eyes
**Age, years (Mean, [SD])**	80.0 (7.6)	–	80.2 (7.6)	79.0 (7.5)	77.7 (7.2)	80.1 (8.4)	81.3 (6.9)	80.1 (7.4)
**Gender**								
**Female: N, %**	49 (57.7)	–	43 (62.3)	10 (38.5)	13 (61.9)	16 (59.3)	15 (65.2)	9 (37.5)
**Male: N, %**	35 (41.2)	–	25 (36.2)	16 (61.5)	7 (33.3)	11 (40.7)	8 (34.8)	15 (62.5)
**Unknown: N, %**	1 (1.2)	–	1 (1.5)	0 (0.0)	1 (4.8)	0 (0.0)	0 (0.0)	0 (0.0)
**VA (ETDRS letters; mean [SD])**	–	64.7 (15.9)	65.2 (14.8)	63.3 (18.9)	81.1 (2.7)	72.6 (2.9)	64.0 (2.3)	42.0 (12.6)
**Injection interval (mean days [SD])**	–	45.7 (23.9)	36.5 (7.5)	70.0 (34.0)	45.7 (17.2)	48.3 (38.2)	45.6 (16.8)	42.8 (12.1)
**Follow up period from first brolucizumab injection (mean days [SD])**	–	642.4 (47.8)	647.2 (48.8)	629.7 (43.5)	653.5 (38.3)	651.4 (58.2)	628.0 (44.3)	636.4 (43.8)
**CMT (N [%])**	–	91 (95.8)	67 (97.1)	24 (92.3)	21 (100.0)	25 (92.6)	21 (91.3)	24 (100.0)
**CMT, µm (Mean [SD])**	–	283.7 (76.2)	286.3 (78.9)	276.5 (69.3)	270.9 (48.3)	284.1 (75.8)	278.2 (72.0)	299.4 (98.8)
**IRF (n [%])**	–	19 (20.0)	14 (20.3)	5 (19.2)	1 (4.8)	3 (11.1)	5 (21.7)	10 (41.7)
**SRF (n [%])**	–	55 (57.9)	35 (50.7)	20 (76.9)	13 (61.9)	15 (55.6)	15 (65.2)	12 (50.0)
**PED (n [%])**	–	55 (57.9)	42 (60.9)	13 (50.0)	9 (42.9)	16 (59.3)	16 (69.6)	14 (58.3)

CMT, central macular thickness; ETDRS, Early Treatment Diabetic Retinopathy Study; IRF, intraretinal fluid; PED, pigment epithelial detachment; SD, standard deviation; SRF, subretinal fluid; VA, visual acuity
