# Supplementary figures and images for: Switching to brolucizumab: injection intervals and visual, anatomical and safety outcomes at 12 and 18 months in real-world eyes with neovascular age-related macular degeneration
Source: Int J Retina Vitreous. 2023 Feb 1;9:8. doi: 10.1186/s40942-023-00445-0 (PMC9891747; doi:10.1186/s40942-023-00445-0)

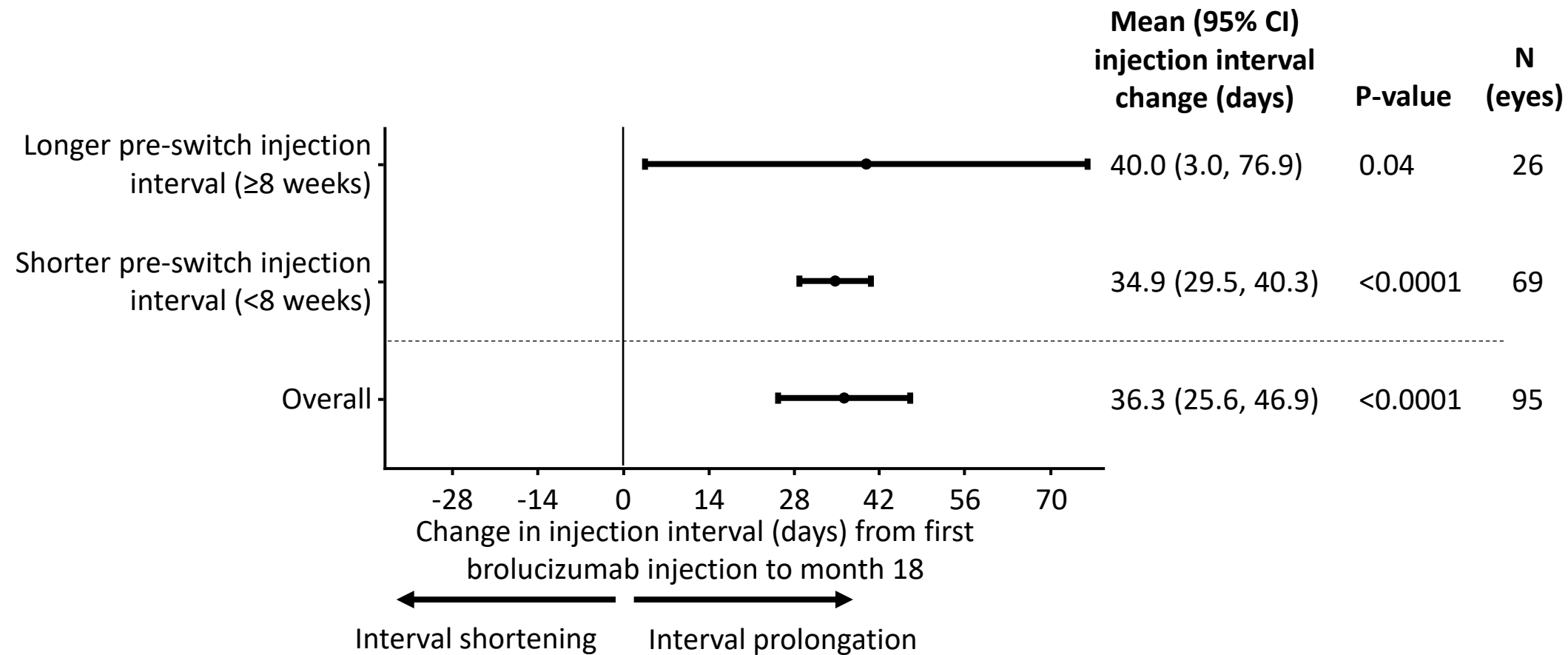

Supplement: Supplementary file 2 — Additional file 2: Figure S1. Effect of baseline injection interval length on injection interval length at Month 18. [file 40942_2023_445_MOESM2_ESM.pdf]

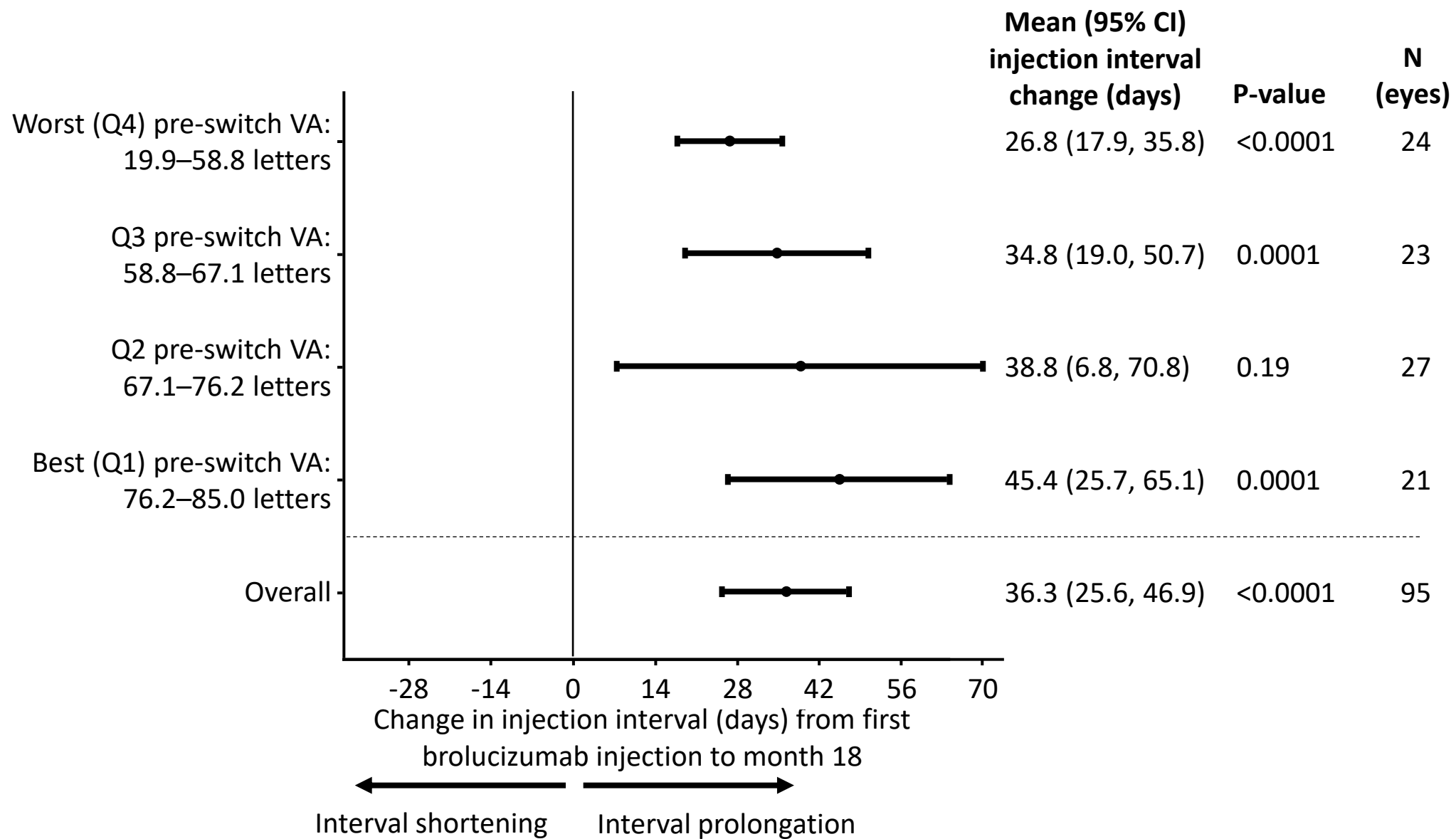

Supplement: Supplementary file 3 — Additional file 3: Figure S2. Effect of baseline VA on injection interval length at Month 18. [file 40942_2023_445_MOESM3_ESM.pdf]

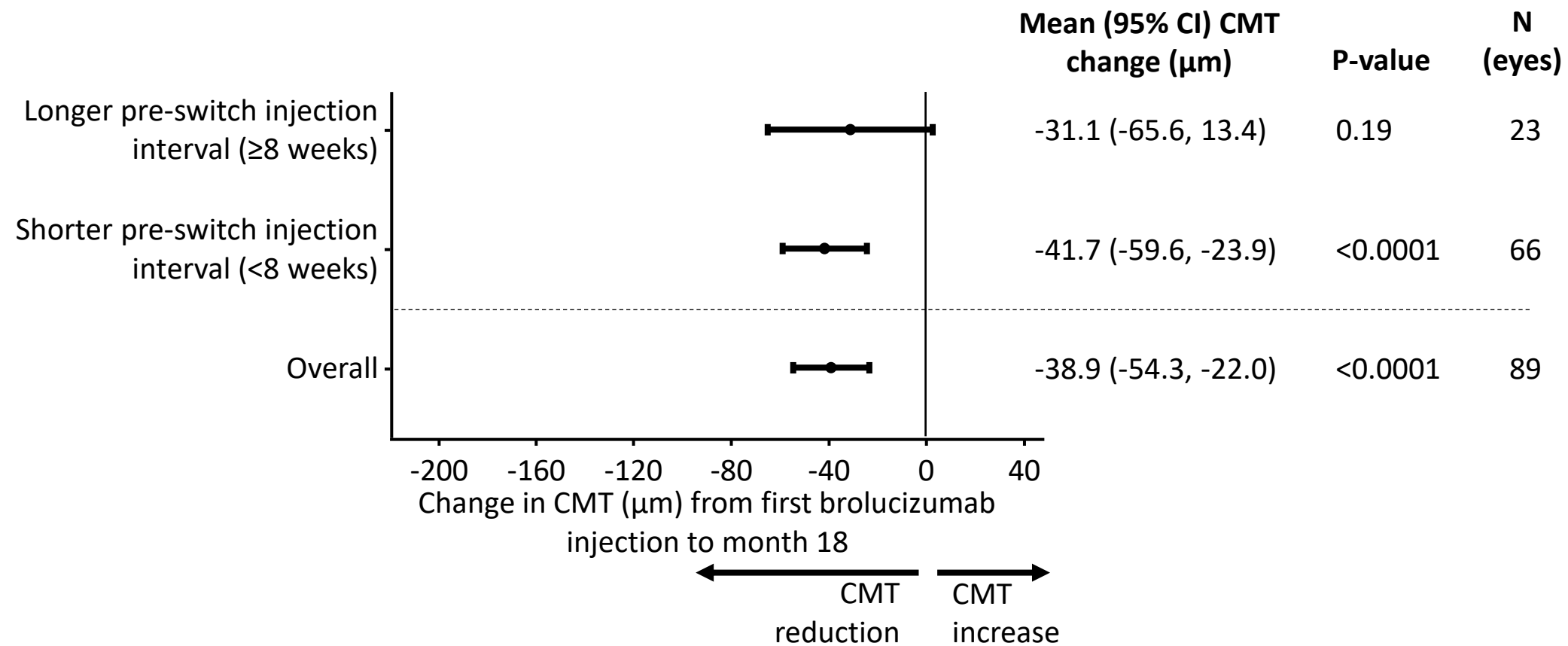

Supplement: Supplementary file 4 — Additional file 4: Figure S3. Effect of baseline injection interval length on CMT at Month 18. [file 40942_2023_445_MOESM4_ESM.pdf]

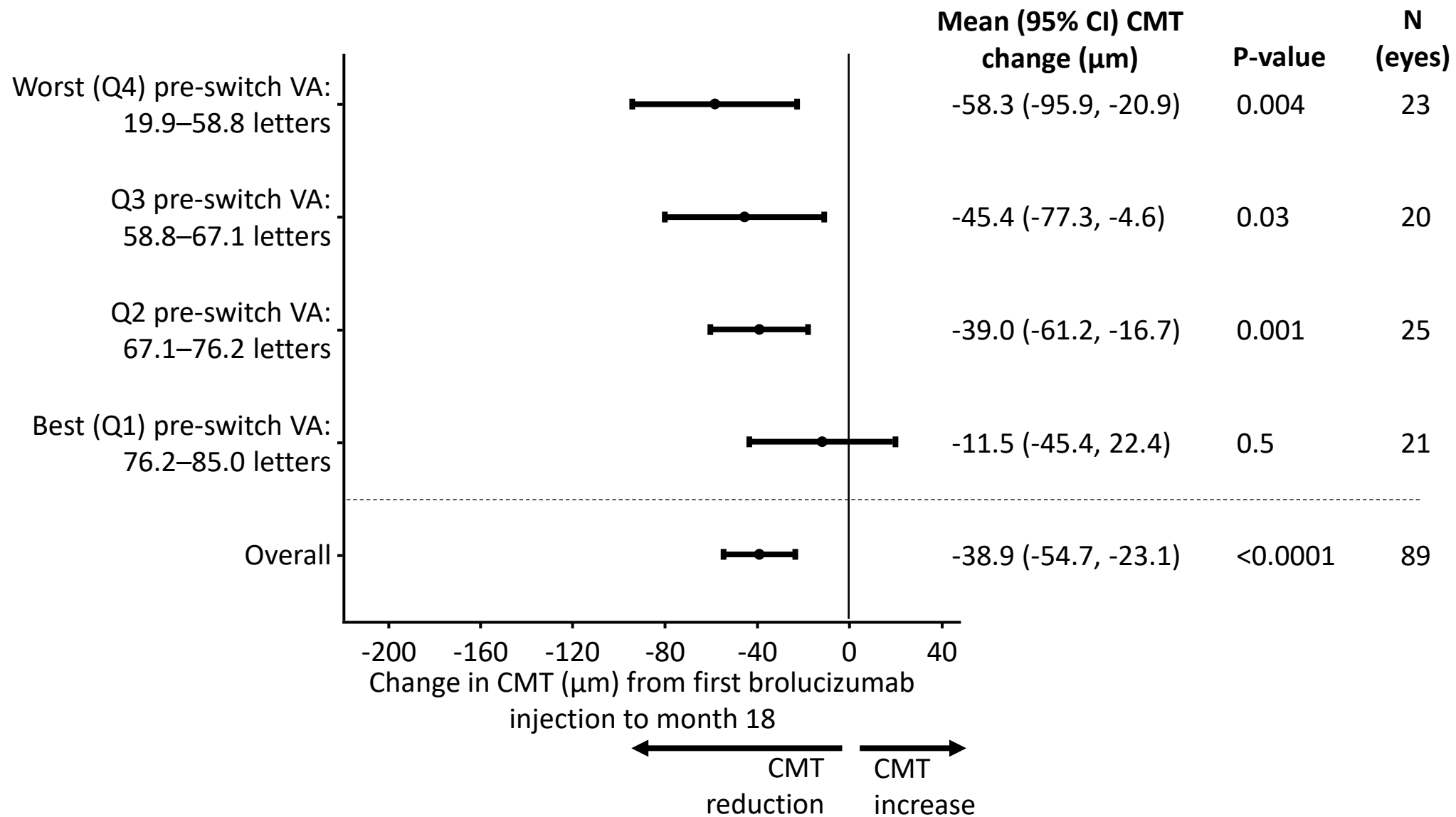

Supplement: Supplementary file 5 — Additional file 5: Figure S4. Effect of baseline VA on CMT at Month 18. [file 40942_2023_445_MOESM5_ESM.pdf]
